# What Makes Intentional Unidirectional Peer Support for Homeless People Work? An Exploratory Analysis Based on Clients’ and Peer Workers’ Perceptions

**DOI:** 10.1177/10497323221083356

**Published:** 2022-03-29

**Authors:** Sandra H. H. Schel, Linda van den Dries, Judith R. L. M. Wolf

**Affiliations:** 1 Impuls - Netherlands Center for Social Care Research, Radboud Institute for Health Sciences, 6034 Radboud University Medical Center, Nijmegen, Netherlands

**Keywords:** intentional unidirectional peer support, homeless people, outcomes, working mechanisms, qualitative interviews

## Abstract

Homeless people are increasingly supported by peer workers in one-on-one mentorship relations called intentional unidirectional peer support (IUPS). Insight is therefore needed into the application and outcome of IUPS for this vulnerable population. This study examined the outcomes, critical elements, and working mechanisms of IUPS within homeless services from the perspective of both clients receiving IUPS and peer workers providing IUPS. Semi-structured face-to-face interviews were held with ten homeless clients and ten peer workers. According to participants, IUPS enhances clients’ self-image, advances their personal growth, and results in better engagement with needed services. Rapport and empathy, a trustworthy and empowering relationship, as well as support, guidance, and mediation are what makes IUPS work according to the participants. The inclusion of both perspectives has led to a deeper understanding of what makes IUPS work for homeless people. Implications for practice are discussed.

## Introduction

Homeless people are increasingly supported by peer workers in one-on-one mentorship relations, called intentional unidirectional peer support (IUPS). Intentional unidirectional peer support refers to “a formalized mentorship type of peer intervention where the peer is clearly more advanced and is mentoring the client in an organized fashion” (Barker et al., 2020). Research investigating the effectiveness of IUPS within homeless populations is scarce (Barker & Maguire, 2017; Faulkner & Basset, 2012). Insight is therefore needed into the application and outcomes of IUPS for this vulnerable population.

Although IUPS in the homeless population is an under-researched area, in this population, several studies on intentional peer support (IPS) in general, have been conducted. Intentional peer support is peer support that is fostered and developed by organizations and can be divided into IUPS and intentional bidirectional peer support (IBPS) which involves reciprocal and mutual types of peer interventions (Barker et al., 2020). One review study assessing all research published between 1995 and 2014 that quantitatively examined the effectiveness of IPS within homeless populations reported that IPS has significant positive impacts on participants’ overall quality of life, drug and alcohol use, mental and physical health, and social support (Barker & Maguire, 2017). However, the results were considered inconclusive, because most studies had substantial methodological limitations, such as a lack of randomization, no control group, or limited power. Until now, the effectiveness of IUPS within homeless populations has only been examined in two qualitive studies, mainly from the peer worker’s perspective. One study assessed the motivation and outcomes experienced by peer workers providing IUPS (Croft et al., 2013). The other study explored the perspective of peer workers and clients with regard to the critical elements that underpin IUPS and also reported outcomes experienced by peer workers (Barker et al., 2018). The two studies reported that the peer workers themselves benefited from helping clients: they experienced empowerment, identity integration, enhanced self-esteem, and confidence as valuable outcomes of IUPS. Outcomes of IUPS for clients were not examined in these studies.

Within populations with mental health problems the effectiveness of IPS has been investigated more often. Intentional peer support has been associated with positive recovery related outcomes, such as hope, empowerment, self-esteem, self-efficacy, self-management, and social inclusion, but these outcomes also need to be interpreted with caution due to the low quality of the research methodologies (Chien et al., 2019; King et al., 2018; Lloyd-Evans et al., 2014; Pitt et al., 2013; Repper & Carter, 2011). Those studies, mostly being randomized controlled trials (RCT, Chien et al., 2019; Lloyd-Evans et al., 2014; Pitt et al., 2013), also generally lack comparability, because the way IPS was provided (for example IUPS vs. IBPS) and the outcomes that were measured varied widely between studies (King et al., 2018). A review assessing qualitative studies on the outcomes of IPS for people with mental health problems, revealed that the majority of outcomes were examined from a peer worker’s perspective (Repper & Carter, 2011). Two qualitative studies that have investigated the outcomes of IPS for people with mental health problems from the clients’ perspective, reported that clients felt better understood by their peer worker than by other professionals (Coatsworth-Puspokey et al., 2006) and noted an increase in community involvement (Trainor et al., 1997).

Besides the notion that the effectiveness of IUPS within homeless populations is still an under-researched area, the utilization of RCTs is being questioned within the field as an appropriate method to investigate peer support (Walker & Peterson, 2021). Outcomes relating to the participant’s long term personal recovery, such as empowerment and resilience, are difficult to grasp with a quantitative questionnaire, and were thus generally not included in previous RCT studies (Walker & Peterson, 2021). By focusing on clients’ and peer workers’ experiences and narratives, qualitative interviews with both these groups can provide this essential in-depth information from each of their perspectives. Qualitative interviews are also useful to comprehend the mechanisms that make IUPS, a complex intervention, work, and induce change (e.g., getting into the “black box” of the intervention) (Greenhalgh & Papoutsi, 2018; Mannell & Davis, 2019; Walker & Peterson, 2021). Through qualitative interviews clients and peer workers can provide insight into the elements that define and are considered critical for IUPS, called critical elements, and the mechanisms establishing a link between these critical elements and the outcomes, shedding light on what makes IUPS work. To the best of our knowledge, there is only one model describing critical elements and potential change mechanisms of IUPS for homeless people (Barker, 2018). This model clarifies three critical elements: (1) The peer-client relationship, referring to the peer worker’s ability to understand the client (e.g., positive regard), the peer worker helping the client to understand that their reactions to oppressive systems are normal (e.g., normalization), the use of experiential knowledge, and empowerment; (2) Role modeling, whereby clients learn from peer workers’ coping techniques and strengths-based approach, where peers advocate for clients and highlight their lived experience as a strength and in which clients evaluate themselves through social comparison with the peer workers; (3) Experience-based social support, whereby peer workers offer different types of support, such as informational support (guiding clients through social services in which the peer worker has already found their way), instrumental support, emotional support, companionship, and appraisal (Barker, 2018; Barker et al., 2019).

Regarding people with mental health problems, studies examining the critical elements and working mechanisms of IPS are less scarce. They do, however, also represent a range of peer roles applied within various mental health care settings, and predominantly involve the perspective of peer workers (Watson, 2019). To our knowledge, only one study within mental health care included the perspective of clients (Gidugu et al., 2015). This study examined the nature and processes of IUPS and what makes IUPS work. It showed that clients particularly benefited from practical, emotional, and social support, valued “having someone to rely on, a friend, and someone to socialize with” and esteemed having a non–treatment-based, normalizing relationship with peer workers. These findings are fairly in line with the critical elements discerned in IUPS for homeless people (Barker, 2018; Barker et al., 2019). Nonetheless, it is evident that homeless people experience additional problems compared to people “only” experiencing mental health problems. Besides physical and mental health problems, homeless people often experience high levels of social exclusion resulting in deprivation in areas such as social relations, material resources, access to health services and housing as well as a lack of future perspective. Therefore, the experiences of homeless people regarding the outcomes, critical elements, and working mechanisms of IUPS should be explicitly assessed.

The purpose of the current study is to extend the previous studies on outcomes, critical elements, and working mechanisms of IUPS with a study that examines those aspects within homeless services from the perspective of both clients receiving IUPS and peer workers providing IUPS. The research questions of this study are threefold: (1) What are the outcomes of IUPS according to homeless people and peer workers? (2) Which critical elements of IUPS do they distinguish? and, (3) Which working mechanisms of IUPS can be identified, based on the perceived outcomes and critical elements for both groups? The results of this study will contribute to the development of an experience-based foundation for IUPS for homeless people and may give clues for the development and maintenance of IUPS.

## Methods

### Design

This study applied a qualitative design, utilizing semi-structured interviews, to examine the outcomes, critical elements, and working mechanisms of IUPS within homeless services, from the perspective of both clients receiving IUPS and peer workers providing IUPS. Face-to-face interviews were held with ten homeless people and ten peer workers.

### Ethical Approval, Quality Criteria, and Research Team

This study was exempt from formal review by the accredited Medical Review Ethics Committee region Arnhem-Nijmegen (file number 2018–5007). All participants gave written informed consent and received verbal and written information about the aim and process of the study, and their right to withdraw at any time, without giving any reason. Participants were aware that their participation was voluntary. At the start of the study, all participants were assigned a research number, ensuring anonymity during the process of data collection, data analysis, and data storage. Only the research team had access to data that could lead to the identification of the participants. The research team consisted of a researcher, a senior researcher, two interviewers, an expert in peer support, and a professor of social care. All were experienced in conducting research and interviewing vulnerable citizens, ensuring the presence of important skills that are required by qualitative health researchers, such as the ability to modify the research methods to fit the contextual and participants limitations, and the understanding of the participants’ situation (Morse, 2010). The interviewers were two psychologists who had not met the participants prior to the start of the study.

### Setting and Participants

In this study, all member organizations of the institution named “Academic Collaborative Center Impulse: Participation and Social Care” that provide ambulant and residential care to homeless people were contacted by the researcher and asked whether they provided IUPS to homeless people and wanted to participate in the study. All five organizations that provided IUPS to homeless people were willing to participate and were included in the study. Four organizations formally employed peer workers and one organization employed peer workers on a voluntary basis. Within two organizations peer workers worked in a team with other peer workers in a special “peer workers department” that was accessible to all clients, for all kinds of support. In the other three organizations, the peer workers worked in a team with a mix of professionals and peer workers where they supported clients alongside the other professionals. Besides IUPS, all five organizations had implemented the strengths-based approach of “Pathways to Empowerment” (Krachtwerk in Dutch; Wolf, 2016) by training and coaching professional staff throughout their ambulant and residential care services. Peer workers were included in this study when they provided support to homeless people in one-on-one relationships. The homeless people were currently receiving this support or had been receiving this assistance within the preceding year. They either lived in transitional or residential care accommodation or had recently moved from such an accommodation to independent (supported) housing in the community.

### Recruitment

Participants were recruited via purposeful sampling. Only Dutch speaking participants were included in this study. Candidate participants who, according to the manager or daily staff, were not able to provide reliable information, for example due to an active psychotic episode, were excluded from this study. Candidate participants received an information letter containing details of the study; peer workers received this letter via their manager and clients via their peer workers. Peer workers who expressed interest in participating were contacted by telephone by the researcher, who provided additional information, answered remaining questions and scheduled the interview. Clients who expressed interest in participating were contacted by their peer worker, who scheduled a face-to-face meeting with the interviewers. Prior to the start of the interview the interviewer provided further information and answered remaining questions. Participants received a twenty-euro gift card as compensation for their time.

### Interview guide and data collection

The interviews were conducted using an interview guide developed by the research team. This guide was based on two sources: expert opinions from the research team and knowledge from scientific literature about IUPS and recovery. After conducting the first two interviews, the interviewers noticed that a few specific questions led to some confusion with the interviewees about the purpose of these questions. This was discussed with the researcher and led to a slight simplification of the interview questions to make them easier to understand. Each interview consisted of two parts: (1) questions about the perceived outcomes of IUPS and (2) questions about the critical elements of IUPS. To examine the perceived outcomes of IUPS, clients were asked whether and how their lives had changed due to the peer support (e.g.,: “How has the peer support affected your life?”). In order to gain insight into the critical elements of IUPS, clients were asked: “What was valuable or important about the support you received from the peer worker?” To help clients talk about their experiences and elaborate on what they perceived as critical elements of IUPS, additional questions were asked, for instance: “What part/element of the peer worker’s support was most helpful to you?” For the peer workers, the questions were adjusted to their role, for example: “What was valuable or important about your support for your clients?” and “How has your support affected your clients’ lives?”

The interviews were held at the location of the participant’s choice, to make participants more comfortable. In the majority of cases, this was a quiet room within the participating organization, to make sure the interviewer and interviewee were not disturbed and to ensure confidentiality. On two occasions, the interview took place at the client’s home or outdoors close to the organization, because the interviewee either had to look after her children or felt uncomfortable being in the allocated room. The interviewer made field notes during and immediately after the interviews. After each interview, the interviewers discussed the findings with the researcher (e.g., peer debriefing) in order to ensure a consistent performance of the interviews, but also to check whether the focus of the interviews was sufficient or should be altered. This did not lead to any changes. Furthermore, after each interview the interviewers reported their observations regarding aspects that could have affected the reliability of the interviews, such as the participants’ functioning, mood, ability to focus and the context in which the interview took place (e.g., location, presence of others). All interviews were considered reliable. The interviews were held between March and July 2019. They took approximately one hour and were recorded with an audio recorder. Audio recordings were transcribed verbatim. To protect participants’ identities, research ID numbers were applied in all transcripts.

### Data Analysis

Data analysis was supported using a qualitative data analysis software program (Atlas.ti version 7). The transcripts of clients and peer workers were independently analyzed using an inductive (“conventional thematic content analysis”) and a deductive (“directed content analysis”) approach as described by Braun and Clarke (2006). To minimize subjectivity of findings, two researchers independently coded the interview transcripts line-by-line, describing the data using initial codes for both the outcomes as well as the critical elements of IUPS. After coding four transcripts, the two researchers met to review and discuss the meaning and uniqueness of the initial codes and agreed upon the initial coding schemes. Then a meeting with a third researcher was organized, during which the initial coding schemes were discussed and consensus about the initial coding schemes was reached. The initial coding schemes formed the basis for the subsequent (selective) coding process. After coding eight, twelve, sixteen, and twenty interviews, the above-mentioned review and consensus process was repeated. After seventeen interviews the transcripts did not generate new codes or modifications to the coding schemes, indicating that code saturation was reached. Interviews eighteen, nineteen, and twenty helped all codes to reach or approach meaning saturation (Hennick et al., 2017). Then, the three researchers discussed and established the final coding schemes of the outcomes and critical elements of IUPS. Next, the researchers extracted, identified and developed possible working mechanisms. During two additional meetings these working mechanisms were discussed and further adjusted.

## Results

The results of this study are reported in terms of outcomes and critical elements of IUPS as experienced and reported by clients and peer workers. Although the interviews with clients and peer workers were independently examined, the results of the analyses were almost identical for these two groups. In this results section, the outcomes and critical elements that were reported by both groups will therefore be referred to as perceived by “participants Regarding the outcomes and critical elements that were mentioned by one group only, this specific group will be indicated in the description of the results and in the Tables. Based on an exploration of the interaction between the perceived outcomes and critical elements this section will also present the working mechanisms of IUPS. First, the characteristics of the participants are described.

### Characteristics of Participants

Of the ten participating clients, five were female, and the average age was 46 years, ranging from 31 years to 56 years, with 50% having a lower and 50% having an intermediate educational level. All clients were single, one person was living with her children. The average duration of lifetime homelessness was 42 months, ranging from 7 months to 10 years. On average, clients had been receiving IUPS for 23 months, ranging from 2 months to 7 years. Eight clients lived in a transitional or residential care accommodation and two clients had recently moved from such an accommodation to independent (supported) housing in the community.

Of the ten peer workers, five were female, and the average age was 42 years, ranging from 26 to 59 years, with 20% having a lower, 70% an intermediate, and 10% a higher educational level. In addition, all peer workers received higher or intermediate professional training in peer support work or had almost finished this training. All peer workers had personal experiences of being homeless at some point during their lives. On average, the peer workers had been supporting (formerly) homeless people for 39 months, ranging from 7 months to almost 10 years. Peer workers were currently providing IUPS to clients at various stages of their recovery process.

### Perceived Outcomes of IUPS

The three perceived outcomes of IUPS were: Positive self-image, Personal growth and Engagement with services, consisting of a total of eight aspects (see Table 1). Below these outcomes are presented, together with participant quotes to give meaning to each outcome.

**Table 1.** Overview of Outcomes of Intentional Unidirectional Peer Support for Homeless People Perceived by Clients and Peer Workers.

Outcomes	Aspects
Positive self-image	^-^ Increased self-worth
	^-^ Increased self-esteem
	^-^ Increased self-respect
Personal growth	^-^ Increased acceptance of self and own situation^a^
	^-^ Progressing in recovery process
	^-^ Feeling more resilient^a^
	^-^ Feeling more positive about the future^b^
Engagement with services	^-^ Engaging with necessary services

^a^Clients only.

^b^Peer workers only.

#### Positive Self-Image

Due to IUPS, many participants reported clients experiencing more self-worth, having more self-esteem, feeling more confident, having more self-respect and being more proud of themselves. A client expressed this by saying: “*Until two years ago I had no self-esteem at all and I had completely lost my self-confidence. My psychologist and peer worker have given me strength again, because of them I regained my self-esteem and self-confidence*.” Peer workers made clients experience that they have a lot to offer, that just being themselves is enough, that they are worthy and can rely on themselves. Peer workers were fully aware of the positive influence their support had on their clients’ self-image. They highly valued this aspect of their profession and considered this the core of what they have to offer their clients.

#### Personal Growth

Participants described that, because of IUPS, clients were able to progress in their recovery process. Clients were taking up things again like looking for a job, starting an internship or following a financial care trajectory. A peer worker exemplified: “*That older man who is off the streets again, has a decent allowance and is able to take part in a debt relief trajectory. He basically has got his whole life back on track and he feels safe and being taken seriously*.” Clients whose daily life used to revolve around drug use for example, developed a more positive perspective on the future due to IUPS and tried to spend their evenings and weekends in a meaningful way. Clients also stated that they felt more resilient and were more able to accept themselves, their current situation and their past due to IUPS.

#### Engagement with Services

Due to IUPS, clients had become engaged with the services they needed. Peer workers helped clients to identify their physical and mental health problems, and to get in touch with the help they needed, for example, from a general practitioner or specialized care. Peer workers also motivated clients to seek, for instance, treatment for their addiction problems, for which the client until then lacked motivation.

### Critical Elements of IUPS

The analysis of the interviews revealed ten critical elements of IUPS, namely: Being there, Understanding, Being accessible and available, Providing comfort and a personal connection, Having an equal relationship, Role modeling, Stimulating and empowering, Providing practical support and developing better life circumstances, Supporting client-professional relationship, and Supporting clients with care trajectories. The elements and aspects—thirty-four in total (see Table 2)—are formulated in terms of actions performed by peer workers. All critical elements are presented below with participant quotes used to give context to elements.

**Table 2.** Overview of Critical Elements of Intentional Unidirectional Peer Support for Homeless People Perceived by Clients and Peer Workers.

Critical elements	Aspects
Being there	^-^ Making contact and show interest in client^a^
	^-^ Providing a listening ear
	^-^ Having plenty of time for client
	^-^ Sincerely caring for client’s well-being
Understanding	^-^ Expressing understanding of client’s situation
	^-^ Deeply understanding the client’s situation, based on shared experiences
	^-^ Acknowledging client’s difficult situation
Being accessible and available	^-^ Being available when client needs it
	^-^ Being accessible after office hours
	^-^ Keeping in touch after client’s trajectory ends
Providing comfort and a personal connection	^-^ Having a special connection with client
	^-^ Keeping less distance (compared to other professionals)^a^
	^-^ Being trustworthy for client, because of shared experiences
	^-^ Actively building a trusting relationship with client^a^
Having an equal relationship	^-^ Accepting client for who he/she is, without being judgmental
	^-^ Treating client as a person and not as a number
	^-^ Putting needs and goals of client first
Role modeling	^-^ Sharing own experiences to help clients^a^
Stimulating and empowering
	^-^ Validating and reinforcing client’s behavior
	^-^ Expressing to clients that they matter
	^-^ Motivating clients to work on their recovery
	^-^ Providing client with informational support about recovery
	^-^ Expressing to client that he/she is responsible for his/her own recovery
	^-^ Confronting client with his/her nonconstructive behavior
Providing practical support and developing better life circumstances	^-^ Providing tips and advice for everyday problems
	^-^ Providing practical support^a^
	^-^ Supporting client with establishing meaningful daily activities
	^-^ Supporting client with forming and strengthening social network
Supporting client-professional relationship	^-^ Mediating between client and professional
Supporting clients with care trajectories	^-^ Supporting client with care trajectories within and outside the organization
	^-^ Supporting client with problems within care trajectories
	^-^ Aiding client by working together with professionals on client’s trajectory^a^
	^-^ Educating professionals about client’s problems^a^

^a^Peer workers only.

#### Being There

Peer workers reported that they established rapport with clients and showed genuine interest in them. This was highly valued by clients, because due to their history of homelessness, not all clients are used to “*someone wanting to have a conversation with them*.” Nearly all participants described the peer worker as providing a listening ear and sincerely caring for clients’ well-being, which made clients feel that they are worthy. This was experienced as a very important aspect of IUPS. Several participants also described that peer workers, unlike some other professionals, had plenty of time to really be there for their clients, which was highly valued. A peer worker exemplified: “*It is just that sometimes, they have to blow off steam and they can do that here…the workload for staff working at the wards often is so high that they do not have time to ‘receive’ that steam from a client. We have a somewhat different set of tasks, so if someone comes to me, or when I go to them, I do have time to ‘receive’ their steam*.”

#### Understanding

According to many participants, clients highly appreciated the peer worker’s deep understanding of the client’s situation because of their shared experiences. Clients sensed that peer workers really know what it is like to have lost everything and to live in a homeless shelter: “*With him it is just like, when I tell him something, he just knows how it is. Because he has experienced it himself*.” Alongside the peer worker’s deep understanding, clients also appreciated peer workers explicitly expressing their understanding and acknowledging the difficult situation clients find themselves in. A client exemplified this: “*And then yes…then I feel down again. But then my peer worker says: ‘Hey, I understand, you don’t want to be here, you don’t belong here. You want to have your own place. Of course, we all see that, and we think you deserve it’*.”

#### Being Accessible and Available

Almost all participants described that clients felt really supported by the fact that the peer worker was available for them when they need him/her: “*It feels like a sort of backup to me, if I have a hard time with something he is always there for me. That you just know from the back of your mind: okay, whatever I run into, there is always someone I can rely on, you know*.” Many participants described that the peer worker was even accessible for clients outside office hours, which reinforced client’s belief that they could truly rely on their peer worker. While it’s not always allowed to keep in touch with clients after they exited the shelter, the peer worker often did so, for example, by sending them WhatsApp messages and visiting them. As a peer worker pointed out: “*I don't think I'm officially allowed to do that, and I don't need to do it, but I want to. I want them to know that I am just there when things are not going well*.”

#### Providing Comfort and a Personal Connection

Nearly all participants reported that clients had a special connection or bond with the peer worker and perceived the peer worker as trustworthy because of shared experiences. Peer workers are able to build a trusting relationship fairly quickly, because they know what they are talking about, due to their own experiences. Peer workers also reported that clients experienced them as keeping less professional distance compared to other professionals, and clients keeping less distance to the peer worker: “*So it’s not only that professionals keep a distance from clients, clients also keep a distance from professionals too, because ‘you never know’, it’s not like the old boys’ network on the street…and I notice that they rather tell their troubles to me.*” Peer workers reported that they were aware of the importance of a safe, trusting and comfortable working relationship with their clients and mentioned that they consciously and actively tried to create such a relationship or bond.

#### Having an Equal Relationship

Participants described that clients appreciated being accepted for who they are and felt the peer worker treated them as equal without judging them, and treated them as a person instead of a number or “just another client.” Participants stated that within this equal and respectful relationship, the needs, and goals of clients were considered as most important by peer workers and were put first. A peer worker elucidated: “*I don't know what it is, just equality. I always say: you are the boss, it is your life*.” Another peer worker mentioned: “*I always said to him that it is very important that you do what is helpful for you…What works for you, that is what you have to do*.”

#### Role Modeling

According to many participants, peer workers served as role models and induced hope in their clients, by showing them that they can get out of their current situation, just like the peer worker did. As a peer worker exemplified: “*I think hope arises, say, at the moment I tell them that I once sat at that table and now everything turned out well…Many people are looking for something to hold onto, especially in times when they feel lost.*” Peer workers also shared their own experiences with clients, for example, of how they handled a specific situation, so clients can learn from them. Peer workers usually shared their experiences only when they thought it would be helpful for their clients. Peer workers elucidated that they consciously and carefully choose what kind of experiences they shared with whom and when. They, for example, tried to pick out the positive things when they told about their recovery and only shared the negative parts over time, when their client was doing better and if it was of added value.

#### Stimulating and Empowering

Many participants described how clients valued the way peer workers validated and reinforced clients throughout their recovery process, by saying things that increased their self-esteem, and providing encouragement to work through their difficulties. Various participants mentioned that peer workers also provided informational support about recovery by advising them, for example, to formulate specific and achievable recovery goals, and to not look too far ahead. Furthermore, participants described that peer workers helped clients to persist and stick with their recovery goals, like a client mentioned: “*Then he says like: ‘Hey, your time will come, you’ll have to bite the bullet just a little bit longer. And stick to your goal, go for it.’ Because well, I’m actually not at the right place now. For me this is actually more of a transitional step to real life again*.” Occasionally peer workers were asked by other professionals in their organization or team to help persuade clients to participate in essential parts of their care trajectory. Participants also described that peer workers tried to activate clients by expressing to the client that they are responsible for their own recovery. Also, some participants mentioned that the peer worker confronted or corrected clients when their behaviors had a negative influence on their well-being and recovery process, as a peer worker stated: “*At a certain point I just confront them and say: ‘Ok, these are the steps you should take, I also made these steps.’ Sometimes you have got to be honest, that’s all part of the game, they’re going to have to bite the bullet.*”

#### Providing Practical Support and Developing Better Life Circumstances

Several participants described that the peer worker advised clients with everyday problems, peer workers also mentioned that they offered clients practical support if needed, such as assistance with their administration. This practical support was still provided after clients had exited the shelter, as a peer worker exemplified: “*I recently helped someone move out. Yes, those things, these are also just part of my work, right... He was standing there obviously with all his belongings and he had to get from A to B.*” Some participants described that the peer worker helped clients establish meaningful daily activities, by exploring their capabilities and preferences regarding hobbies, work or other activities. Some participants mentioned that peer workers also supported clients with forming and strengthening their social network.

#### Supporting Client-Professional Relationship

Several participants mentioned that the peer worker acted as a liaison and mediator between the client and his professional caregiver, for instance, by trying to explain to a colleague how a client sees something or vice versa. When problems arose within the relationship between the client and his professional caregiver, peer workers often helped clients to solve these problems: “*We receive a lot of complaints from clients about professionals. So, when we receive a complaint, first we look at what is exactly going on and then we try to mediate between both parties. This usually results in the complaints being settled*.”

#### Supporting Clients with Care Trajectories

Most participants felt that peer workers supported clients with all kinds of issues associated with their care trajectories within and outside their organization. They helped clients for example with navigating the social care and mental health system to get them the services they needed, finding treatment for physical problems or applying for housing. Many participants reported that peer workers helped clients to solve problems that arose within their care trajectories and that they aided clients by working together with other professionals on a clients’ trajectory: “*When regular professionals run into a problem with their client, they ask us (peer workers) to assist within the client’s care trajectory or to work alongside them when this problem is being discussed within a team meeting*.” Peer workers described that they were actively involved in educating professionals about certain difficulties or mental health problems that clients experience, such as addiction or self-mutilation, in order to create a greater understanding among professionals of how to effectively support clients with these problems.

### Working Mechanisms of IUPS

**Figure 1.**
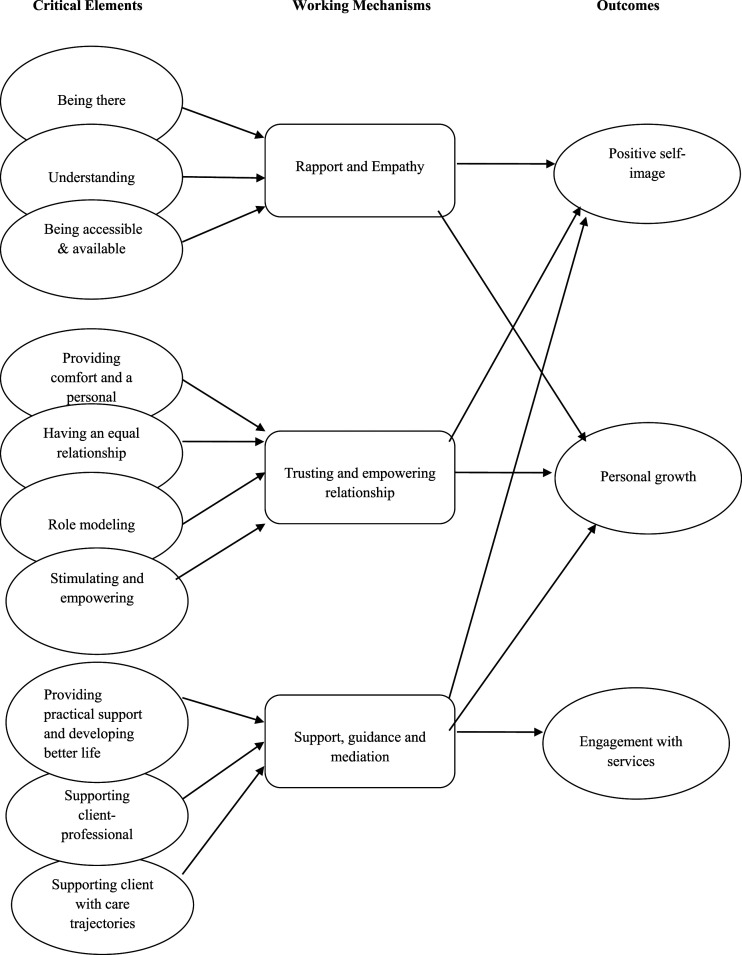
Outcomes, critical elements, and working mechanisms of intentional unidirectional peer support in homeless services, based on clients and peer workers experiences.

Based on an exploration of the interaction between the above-mentioned outcomes and critical elements of IUPS as perceived by homeless people and peer workers, three mechanisms were identified that make IUPS work for homeless people: Rapport and empathy, Trusting and empowering relationship and Support, guidance and mediation (Figure 1).

#### Rapport and Empathy

Because peer workers build rapport, offer a listening ear and sincerely care for clients’ well-being, the clients feel they are being taken seriously and appreciated, and that an effort is being made for them. These feelings are reinforced by the fact that peer workers often have ample time for clients and are available outside office hours. Peer workers deeply understand the difficult situation the clients are in, and by sharing their experiences with clients consciously and carefully they validate and empower clients’ experiences and needs. Explicitly acknowledging the client’s difficult situation and the challenges in their process of recovery further validates their experiences. The genuine and reciprocal interaction between the two increases clients’ self-esteem, self-worth and self-respect, which also leads to more self-acceptance and acceptance of their own situation.

#### Trusting and Empowering Relationship

Because peer workers actively work on establishing a trusting and secure relationship and keep less “distance” from clients compared to other professionals, clients tend to confide in peer workers rather quickly, open themselves up and share their experiences with peer workers. Being treated as an equal person without being judged and not being treated as “a number” underpins this trusting and secure relationship as well. This relationship encourages clients to regain hope, set small goals, and work towards realizing them. Their trust in the peer worker, based on having shared experiences, also makes clients take on the peer workers’ advice quickly. They accept peer workers being critical and addressing and confronting their nonconstructive behavior. Peer workers also express to clients that they matter, they actively motivate clients to work on their recovery and further empower them by providing clients with informational support about recovery. This comforting and nurturing relationship also provides possibilities for self-reflection and learning, and stimulates and motivates clients to grow and progress in their recovery process step by step and day by day. The fact that peer workers put clients’ needs and goals first helps herein as well. Peer workers also function as role models providing clues as to how to go about certain challenges and showing that things could actually turn out well in the end, generating a more positive perspective on the future.

#### Support, Guidance and Mediation

In order to develop better life circumstances for clients and to let them progress in their recovery process, peer workers provide clients with all sorts of practical support, such as tips and advice for everyday problems, support with establishing meaningful daily activities and support with strengthening their social network. The knowledge that they are no longer alone in life and that they can turn to the peer worker for help and advice with all sorts of problems, eases the difficulties they face during their recovery process, and makes clients feel more resilient, more self-confident, and more positive about their future. Supporting clients with their care trajectories and with their relationships with other professionals, also makes clients engage with services they need. The peer workers’ own experiences with and knowledge of care trajectories enables them to provide clients with useful advice and support as regards their contact with professionals and organizations. When clients experience problems within care trajectories or with other professionals, peer workers act as mediators between clients and professionals, making sure that clients obtain and maintain the right care. In order to maintain and improve the quality of care for clients, peer workers also educate other professionals about clients’ problems and advice and assist other professionals in working on clients’ care trajectory.

## Discussion

This is the first study to qualitatively examine the outcomes, critical elements and working mechanisms of IUPS for homeless people from the perspective of both clients and peer workers. Both groups mentioned the same outcomes and critical elements of IUPS. However, certain aspects of outcomes and critical elements were only mentioned by a single group. The inclusion of both perspectives has therefore provided a more complete understanding of the identified outcomes and critical elements of IUPS.

Regarding the outcomes of IUPS, the findings of this explorative study show that homeless people and peer workers perceive a more positive self-image, personal growth and better engagement with services as valued outcomes of IUPS. For the outcome personal growth, the aspects increased acceptance of self and own situation and feeling more resilient were only mentioned by clients and the aspect feeling more positive about the future was only pointed out by peer workers. Until now, there was insufficient knowledge about the outcomes of IUPS for homeless people, because, as was mentioned in the introduction, most previously conducted quantitative studies described various types of IPS and had substantial methodological limitations (Barker & Maguire, 2017). Furthermore, the few qualitative studies that have been carried out did examine the outcomes of IUPS (Barker et al., 2018; Croft et al., 2013), but only from the peer worker’s perspective and did not, as in this study, examine the outcomes for clients and from both perspectives. Nevertheless, the aspects of the outcomes of IUPS for homeless people that we identified in our study are much in line with the outcomes found for peer workers in homeless settings, namely, perceived empowerment, identity integration, enhanced self-esteem, and confidence (Barker et al., 2018; Croft et al., 2013). The findings also compare well with the positive recovery related outcomes found among people with mental health problems, such as increased hope, empowerment, self-esteem, self-efficacy, self-management, and feeling better understood by the peer worker but differ from the reported outcomes regarding increased social inclusion and increase in community involvement (Chien et al., 2019; King et al., 2018; Lloyd-Evans et al., 2014; Pitt et al., 2013; Repper & Carter, 2011).

This study also identified critical elements of IUPS: Being there, Understanding, Being accessible and available, Providing comfort and a personal connection, Having an equal relationship, Role modeling, Stimulating and empowering, Providing practical support and developing better life circumstances, Supporting client-professional relationship and Supporting client with care trajectories. For the critical elements, Being there, Providing comfort and a personal connection, Role modeling, Providing practical support and developing better life circumstances, and Supporting client with care trajectories certain aspects were only mentioned by peer workers. Regarding Providing comfort and a personal connection for example, the aspects Keeping less distance (compared to other professionals) and Actively building a trusting relationship with client were solely described by peer workers. Concerning the critical element Supporting client with care trajectories for instance, peer workers only mentioned the aspects Aiding client by working together with professionals on client’s trajectory and Educating professionals about client’s problems.

The critical elements found in our study seem to coincide with critical elements revealed by peer workers providing IUPS within homeless services as studied by Barker (2018) who used slightly different terms and categorizations: (1) Peer-client relationship; (2) Role modeling; and (3) Experiences based support (Barker, 2018; Barker et al., 2019). In addition to this earlier research, the current study found that participants explicitly, and highly, valued peer workers being present—Being there—and within easy reach—Being accessible and available. The fact that those elements seem rather obvious and basic in providing support to clients, might have been a factor that could explain why Barker (2018) did not identify these elements as separate critical elements of IUPS.

By combining the three outcomes and the ten critical elements as perceived by clients and peer workers, three working mechanisms of IUPS emerged: Rapport and Empathy, Trusting and Empowering Relationship and Support, Guidance and Mediation. The results demonstrate in particular that the quality of the relationship between clients and peer workers is vital to make IUPS work. These results are much in line with previous research about the processes of IUPS within mental health care, showing that clients particularly benefited from practical, emotional, and social support, valued “having someone to rely on, a friend, and someone to socialize with” and esteemed having a non–treatment-based, normalizing relationship (Gidugu et al., 2015). The findings of this study also underline the importance of applying strengths-based approaches such as “Pathways to Empowerment” (Wolf, 2016) that emphasize, for example, the importance of focusing on the client’s strengths and assets, building a trusting relationship with them, supporting the client in her/his process of recovery, and taking the client’s needs and goals as a starting point for the support provided.

### Strengths and Limitations

This study has several strengths. First, to our knowledge, this is the first study that examined the outcomes and the critical elements of IUPS for homeless people from the perspective of the clients as well as the perspective of the peer worker and compared both insights. Additionally, the interaction between the outcomes and critical elements of IUPS were explored, resulting in three mechanisms that make IUPS work for homeless people. These findings contribute to the still under-researched area of IUPS within homeless populations and provide an experience-based grounding for IUPS with clues for its development and improvement. Another strength of this study is the rigor of the data collection and data analysis. For example, the following procedures were applied: anonymous transcription of each interview, making field notes after each interview, thick description, in-depth and independent data analysis by two researchers and peer review and debriefing with the senior researcher and the professor. This significantly increased the reliability of our findings.

This study has several limitations as well. First, we conducted a fixed number of interviews (ten clients and tenpeer workers) and did not have the possibility to interview more people, which is not ideal reaching code and meaning saturation. In this study, code saturation was reached at seventeen interviews, but it was not always clear whether meaning saturation was reached. Previous research found that high prevalence codes generally reach meaning saturation at around nine interviews and that low prevalence codes mostly require between sixteen and twenty-four interviews (Hennick et al., 2017). This implies that the twenty interviews performed in this study should have been sufficient in reaching meaning saturation for most codes. In order to be sure of meaning saturation, future research should not start the study with a fixed number of interviews but should leave the number of interviews open until this has been reached for all codes. Second, clients were recruited by their peer worker. This might have led to some selection bias, for example resulting in a more positive perspective on IUPS when clients with high appreciation for their peer worker or clients who have benefited relatively much from IUPS may have been more willing to participate. Also, clients who, from the viewpoint of the peer worker, were considered to be less able to reflect on their personal experiences may not have been asked to participate in this study. It is unknown if selection bias, perhaps also for other reasons, have occurred. Despite these potential limitations, the rigor of the data collection and data analysis strengthen the credibility and value of the results.

To increase the transferability of the findings, a variety of residential care services throughout The Netherlands were included, such as generic residential care services for homeless people and residential care services specifically aimed at homeless people with addiction problems and severe mental health problems. However, the results of this study might not be entirely applicable to IUPS provided in other types of homeless services, such as emergency shelters or night shelters, as homeless people residing in such accommodations might have other or additional support needs. Also, because this study specifically examined IUPS, the findings are not expected to fully match other forms of peer support within homelessness services, such as peer support groups led by peer workers. Moreover, all peer workers included in this study had received higher or intermediate professional training in peer support work or had almost finished this training. This implies that IUPS examined in this study was of a certain quality that is not necessarily comparable with other forms of IUPS, such as IUPS provided by peer workers who had not received professional training in peer support work or peers providing mutual support.

### Implications for Practice

The findings of this study could help homeless services to optimize IUPS in order to make it work for homeless people. This study shows IUPS for homeless people is greatly valued by clients and peer workers, indicating that IUPS could be considered an important addition to the spectrum of care provided by professionals. Implementing IUPS as standard care thus would further advance care provision within homeless services, provided that organizations will ensure that IUPS is effectively implemented within their services. Organizations should for example use a clear job description with relevant competencies for peer workers providing IUPS and ensure that they are able to use these competencies (which they have obtained through their life experiences and training), instead of being assigned tasks that other professionals are too busy to perform (such as providing clients with transportation when they move out; Davidson et al., 2012). Furthermore, the results of this study suggest that organizations should make sure that peer workers have and retain ample time to “be there” for clients, for instance by preventing high caseloads for peer workers. Organizations should also formally allow and support peer workers being available for clients after office hours or after clients have exited a shelter, if they think this could benefit clients. At the same time, organizations should assist peer workers in maintaining a sound work-life balance, as research shows that role and time boundaries can be a concern for peer workers providing and clients receiving peer support (Gidugu et al., 2015; Miyamoto & Sono, 2012). Additionally, reducing caseloads for other professionals might be beneficial for clients as well, as our results show that the clients feel that other professionals are often too busy to just be there for them and therefore highly value peer workers having ample time for them. When other professionals have more time available for their clients, this could lead to clients feeling listened to and valued as a human being.

The results of this study also suggest that clients could benefit from grounding service provision within homeless services more on an equal footing. Organizations could for example make all professionals aware of the importance of a trusting and equal working relationship and could specifically encourage other professionals to act in a less reserved way when it comes to sharing their personal experiences with clients. This approach, however, is still quite unlike current daily practice within care services for homeless people. During their professional training and in the workplace, professionals are taught to not disclose their personal experiences to clients, while sharing these experiences could in fact be beneficial for clients (Weerman, 2016). However, like peer workers in the current study already indicated, professionals should only share their personal experiences when they think this would be beneficial for clients.

This study showed that peer workers are involved in educating professionals about certain challenges that clients face in their process of recovery and are asked to inform and aid other professionals in dealing with these challenges. This suggests the value of experience-driven service provision and the need among other professionals to learn more about what it takes to recover and what challenges clients are confronted with within this process.

## Conclusion

Homeless people and peer workers greatly value the outcomes and workings of IUPS. Rapport and empathy, a trustworthy and empowering relationship, and support, guidance and mediation are what makes IUPS work. Intentional unidirectional peer support in homeless services enhances client’s self-image, advances their personal growth and results in better engagement with needed services. Intentional unidirectional peer support should therefore be considered standard support in service provision for the homeless.
